# Therapeutic Efficacy of Valproic Acid in a Combined Monocrotaline and Chronic Hypoxia Rat Model of Severe Pulmonary Hypertension

**DOI:** 10.1371/journal.pone.0117211

**Published:** 2015-01-28

**Authors:** Beidi Lan, Emiko Hayama, Nanako Kawaguchi, Yoshiyuki Furutani, Toshio Nakanishi

**Affiliations:** Department of Pediatric Cardiology, Tokyo Women’s Medical University, Tokyo, Japan; University of Giessen Lung Center, GERMANY

## Abstract

**Background:**

Pulmonary hypertension (PH) is a serious disease with poor prognosis. Reports show that cells in remodeled pulmonary arteries of PH patients have similar characteristics to cancer cells, such as exuberant inflammation, increased proliferation, and decreased apoptosis. An ideal strategy for developing PH therapies is to directly target pulmonary vascular remodeling. High levels of histone deacetylase (HDAC) expression and activity are found in certain cancers, and research has shown the potential of HDAC inhibitors in repressing tumor growth via anti-inflammatory and anti-proliferative effects. To date, little is known about the effectiveness of HDAC inhibitors against pulmonary vascular remodeling in severe PH.

**Objective:**

To investigate whether class I HDAC inhibitors suppress or reverse the development of severe PH in rats.

**Methods:**

Male Sprague-Dawley rats were injected with a single, subcutaneous dose of monocrotaline (60mg/kg), and were exposed to chronic hypoxia to induce severe PH. Valproic acid, a class I HDAC inhibitor, was administered to rats daily via gastric gavage (300mg/kg) in a PH prevention study (during the first 3 weeks) or a PH reversal study (from 3 to 5 weeks). At the end of experiment, hemodynamic indices were measured, ventricular hypertrophy indices were calculated and vascular remodeling phenotypes were analyzed. Results: After 3 weeks exposure to a combined stimulation of monocrotaline and chronic hypoxia, rats exhibited a reduced body weight, elevated right ventricular systolic pressure, an increased Fulton index, right ventricle weight ratio, medial wall thickness and muscularized peripheral pulmonary arteries. These parameters for PH evaluation were exacerbated from 3 to 5 weeks. Daily administration of valproic acid therapy prevented and partially reversed the development of severe PH in rats, and decreased inflammation and proliferation in remodeled pulmonary arteries.

**Conclusion:**

These data show that class I HDAC inhibitors may be effective for treating severe PH.

## Introduction

Pulmonary vascular remodeling is a prominent feature of pulmonary hypertension (PH), and gradually leading to increased vascular resistance and right ventricular hypertrophy (RVH). Advanced progression of PH usually causes life-threatening right ventricular failure. Cells in remodeled pulmonary arteries (PA) of PH patients were reported to share similar characteristics with cancer cells, such as exuberant inflammation, increased proliferation, and decreased apoptosis [1]. To date, therapies to efficiently suppress pulmonary vascular remodeling in PH remain to be established.

Recent studies have focused on the role of histone deacetylases (HDACs) in epigenetic regulation of cancer development. Histone acetyltransferases and HDACs regulate genes transcription by modulating histone acetylation and deacetylation. High levels of HDACs expression and activity have been reported in certain cancers, and recent studies highlight the potential of HDACs inhibitors to repress tumor growth via anti-inflammatory and anti-proliferative effects [2–3]. Based on these studies, we hypothesized that HDACs inhibitors may also be effective against pulmonary vascular remodeling associated with PH. However, therapeutic use of HDACs inhibitors has not been extensively investigated in PH. Valproic acid (VPA) is commonly used as a high tolerance/low toxicity antiepileptic agent, and it also is shown to inhibit class I HDACs activity. VPA has been investigated as a potential cancer therapy due to its role in suppressing cell proliferation and inflammation [4]. Benefits of VPA therapy in a hypoxia-induced PH rat model with mild developed pulmonary vessel remodeling has also been reported [5].

In this study, we tested whether HDACs inhibition by VPA attenuates the progression of pulmonary vascular remodeling by reducing excessive inflammation and cell proliferation in a rat model of severe PH. To induce severe PH in rats, we developed a combination method consisting of a single, subcutaneous injection of monocrotaline (MCT) in conjunction with exposure to chronic hypoxia (CH). We then examined the effects of VPA therapy in this severe PH model, and gained important insight into the underlying molecular mechanisms regulating pathological vascular remodeling associated with PH.

## Methods

### Experimental animals and PH induction protocols

All experimental procedures performed in animals were conducted from a protocol approved by the Institutional Animal Experiment Committee of the Tokyo Women’s Medical University.

All procedures were performed under isoflurane-induced, inhalational anesthesia to minimize suffering. To compare differences in the development of PH, Male Sprague-Dawley rats (280–350 g; Tokyo Experimental Animal Company, Japan) (n = 6/group) were randomly assigned to one of the following treatment groups for 3 weeks: (1) Control; rats were injected with saline and maintained in a normoxic chamber. (2) CH; rats were injected with saline and maintained in a hypoxic chamber. (3) MCT; rats were injected with a single dose of MCT and maintained in a normoxic chamber. (4) MCT/CH; rats were injected with a single dose MCT and maintained in a hypoxic chamber [6]. Nine additional MCT/CH rats were histologically analyzed at the end of 3 (MCT/CH 3w), 4 (MCT/CH 4w), and 5 (MCT/CH 5w) weeks (n = 3/group) to determine the progression of severe PH at different time points.

MCT (Sigma Aldrich, St. Louis, USA) was dissolved in 1N HCl, neutralized with 1N NaOH, and diluted with distillated water to 6 mg/mL. A dose of 60 mg/kg was administered to animals in the MCT and MCT/CH groups on study day 1 via subcutaneous injection in the cervical region. Hypoxic conditions were created by continuously flushing a gas mixture (low %O_2_ and high %N_2_) into a ventilated chamber. The oxygen concentration was maintained to 10% [7]. All rats has unlimited access to food and water and were weighted weekly.

In the prevention study, MCT/CH rats (n = 6) received Vehicle or VPA 300 mg/kg daily for 3 weeks. In the reversal study, MCT/CH rats (n = 6) were treated daily with the Vehicle or VPA 300 mg/kg from 3 to 5 weeks. VPA was dissolved in Milli-Q water (30 mg/mL), and rats were gavaged by VPA 1 mL/100 g body weight. The VPA dose used in this study was similar to that administered by Zhao et al. [5].

### Hemodynamic and right ventricular hypertrophy evaluation

The mean systemic blood pressure (SBP) of each rat was measured by a non-invasive tail blood pressure system (BP-98A-L; Softron, Tokyo, Japan). After the SBP value was recorded, we anesthetized the rats by isoflurane inhalation, and exposed the right jugular vein by blunt dissection. Instead of pulmonary arterial pressure, right ventricular systolic pressure (RVSP) was measured by inserting a micro-tip catheter (Millar Instruments, Houston, USA) from the right jugular vein into the right ventricle through a pre-inserted, 18-G tube (Terumo, Tokyo, Japan). Physical signal data was recorded and analyzed by a PowerLab Data Acquisition system and Lab Chart 7 software (ADInstruments, Dunedin, New Zealand). After hemodynamic measurements were obtained, rats were killed by cervical dissection. Heart chambers were harvested and weighted separately as (1) the free wall of the right ventricle (RV), and (2) the left ventricle and septum (LV+S) to evaluate RVH. The Fulton index (weight ratio of RV/ (LV+S)) and RV/ BW (the weight ratio of RV to total body weight) were calculated. Lungs were collected for morphologic and quantitative analysis.

### Quantitative analysis of pulmonary vascular remodeling

Rat lungs were fixed by tracheal infusion with 4% paraformaldehyde (PH = 7.4) and were then immersed in 4% paraformaldehyde solution overnight. Paraffin-embedded lung tissue was cut into 4-μm-thick sections. Victoria blue staining was used to show the elastic lamina (light blue). Immunohistochemical analyses were conducted using α-smooth muscle actin (SMA) (1:200; Sigma), fetal liver kinase 1 (FLK1) (1:50; Santa Cruz, Dallas, USA), ED1 (CD68: anti rat monocytes, macrophages, and dendritic cells) (1:100; BMA Biomedicals AG, August, Switzerland), proliferating cell nuclear antigen (PCNA) (1:125; Sigma), and cleaved caspase 3 (1:100 Cell Signaling Technology, Davers, USA) primary antibodies, followed incubation with a biotinylated secondary antibody and avidin-biotin complex (Vector Laboratories, Peterborough, UK). Mayer hematoxylin was used for counterstaining.

Lung sections were stained with SMA to determine medial wall thickness (%MT) of the PA. Only intra-acinar arteries with a complete muscular coat were measured. The external diameter (ED) and medial wall thickness were measured in 25 muscularized PAs (ranging in size from 20–50μm in external diameter). The %MT was calculated by the following formula: %MT = (2MT/ED)×100 [8]. SMA stained sections were also analyzed for the muscularization ratio of pulmonary vessels. Each vessel was categorized as non-muscularized (no evidence of SMA staining) or muscularized (SMA identified in all or part of vessel circumference). For each rat, the proportion of non-, partially-, and fully-muscularized small PAs (20–50μm) was assessed by counting the number in each category. Vascular occlusion was assessed by the percentage of 50 intra-acinar PAs in 3 lung tissue samples per group, and it was categorized as Grade 0 (no evidence of neointimal formation), Grade 1 (less than 50% luminal occlusion), or Grade 2 (more than 50% luminal occlusion) [9]. PCNA-positive cells in PAs were counted in 30 vessels per rat, and the number of PCNA-positive cells per pulmonary vessel was used as an index of proliferation. The same method was performed in ED1-positive cells [10].

### Western blotting

Lung tissue protein was extracted by homogenizing samples in radioimmunoprecipitation assay buffer (Sigma) containing a protease inhibitor (Roche, Basel, Switzerland) and a phosphatase inhibitor (Roche). Nuclear protein was isolated using a nuclear extraction kit (Affymetrix Panomics, Fremont, USA). Protein concentration was determined with a Coomassie blue-based protein quantitation method (Bradford Ultra; Novexin, Cambridge, UK). Extracted protein (5–10 μg) was resolved with sodium dodecyl sulfate-polyacrylamide gel electrophoresis and transferred to a polyvinylidene fluoride membrane (Hybond-P; GE Healthcare, Waukesha, USA). Primary antibodies to HDAC1, HDAC2, HDAC3, acetylated histone 3 (1:1000; Millipore-Upstate, Temecula, USA) and lamin A/C (1:1000; Santa Cruz) were used, and then membranes were incubated with a horseradish peroxidase-conjugated secondary antibody. Immunoblot signals were developed by incubation with chemiluminescent horseradish peroxidase substrate (Millipore-Upstate) and detected by lumino-analyzer (LAS-4000 mini; Fujifilm, Tokyo, Japan). The expression levels of target proteins were normalized to β-actin or Lamin A/C.

### Real-time RT PCR analysis

Total RNA was isolated from the pulmonary artery (PA) (diameter ranging from 400–1100μm) excised from the right lower lobe of the lung by a RNeasy Mini Kit (QIAGEN, Amtsgericht Düsseldorf, Germany). First-strand complementary DNA was synthesized with Superscript VILO MasterMix (Invitrogen, Carlsbad, USA). The reaction mixture was incubated at 25°C for 10 min, at 42°C for 1 h, and then terminated at 85°C for 5 min. Real-time reverse transcription-polymerase chain reaction (RT-PCR) primers were designed using Roche online software (Universal Probe Library) as shown in Table 1. The signal of each band was calculated and normalized to β-actin.

**Table 1:** Rat primers used for RT-PCR analysis.

Gene	Forward primer	Reverse primer
Casp3	5’-CGTGAAGAAATTATGGAATTGATG-3’	5’-TTCATCTCCATGACTTAGAATCACA-3’
Bcl2	5’-GTACCTGAACCGGCATCTG-3’	5’-GGGGCCATATAGTTCCACAA-3’
Bcl-xl	5’-TGACCACCTAGAGCCTTGGA-3’	5’-TTCCCGTAGAGATCCACAAAA-3’
p21	5’-GACATCTCAGGGCCGAAA-3’	5’-GGCGCTTGGAGTGATAGAAA-3’
MCP1	5’-AGCATCCACGTGCTGTCTC-3’	5’-GATCATCTTGCCAGTGAATGAG-3’
HIF1a	5’-CAAAGACAATAGCTTTGCAGAATG-3’	5’-ACGGTCACCTGGTTGCTG-3’
β-actin	5’-CTAAGGCCAACCGTGAAAAG-3’	5’-GCCTGGATGGCTACGTACA-3’

*Bcl2*, B-cell lymphoma 2;

*Bcl-xl*, B-cell lymphoma-extra large;

*MCP1*, monocyte chemoattractant protein 1;

*HIF1a*, hypoxia induced factor 1a.

### Statistical analysis

A p value of <0.05 was considered statistically significant. Quantitative data from all groups were presented as the mean ± the standard deviation. Data were analyzed with one-way analysis of variance.

## Results

### The combination MCT/CH method increased PH severity in rats compared to single methods

**Fig 1:**
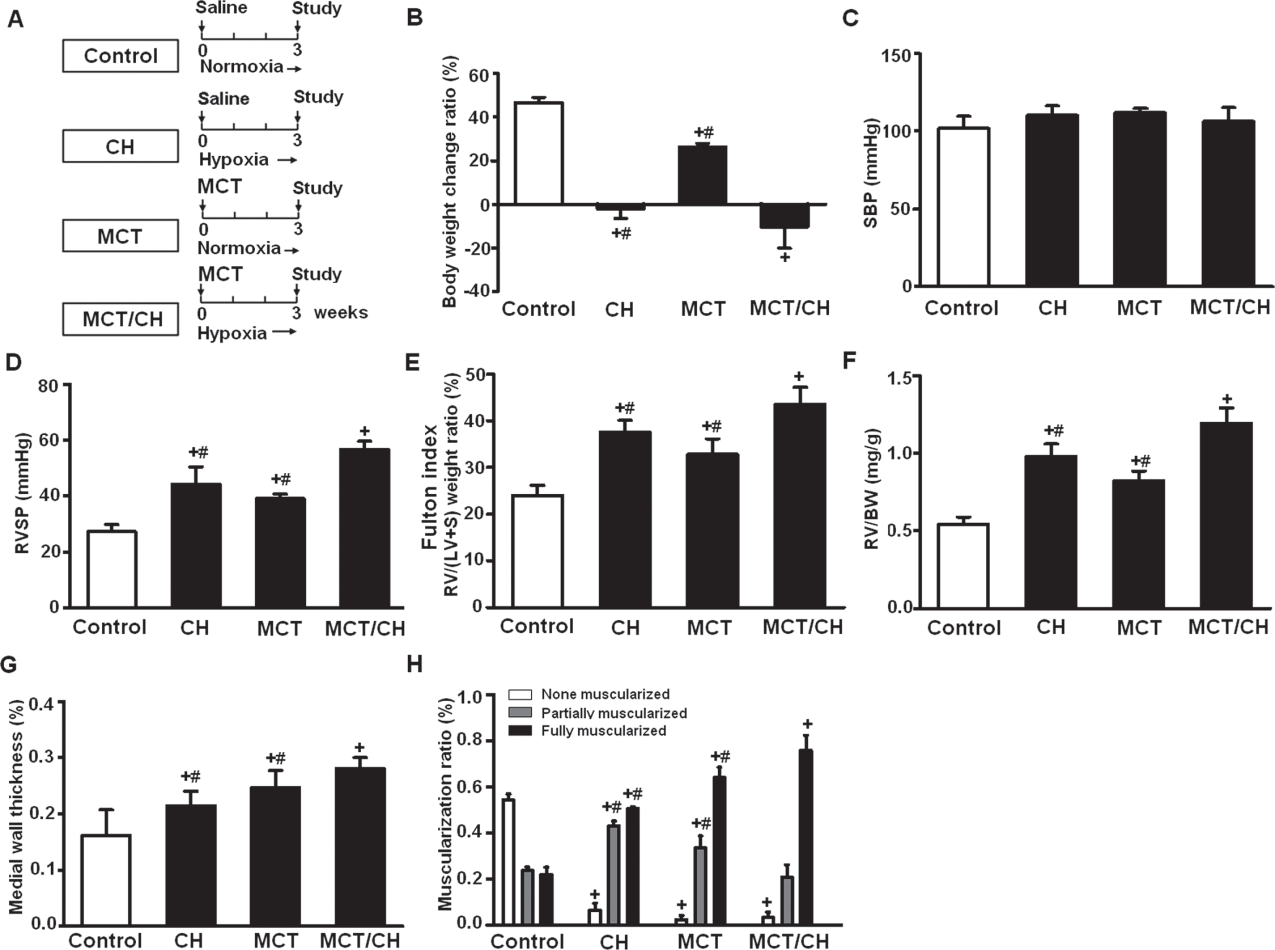
A combination method increased severity of pulmonary hypertension (PH) in rats. (A) Schematic PH model protocols for control, chronic hypoxia (CH), monocrotaline (MCT), and combination (MCT/CH) groups. Comparison of (B) body weight change ratio, (C) systemic blood pressure (SBP), (D) right ventricular systolic pressure (RVSP), (E) Fulton index, (F) the ratio of right ventricle weight to body weight (RV/BW), (G) medial wall thickness and (H) muscularization ratio of small pulmonary arteries among different treatment groups (n = 6 per group). + p < 0.05 vs. control group; # p < 0.05 vs. MCT/CH group.

The development of PH was compared among 3 methods: CH, MCT and MCT/CH (n = 6 /group; Fig. 1A). Three weeks after the start of the experiment, rats in the control group were active and gained weight gradually, whereas rats in the three PH model groups lost weight, with the most significant weight loss occurring in the MCT/CH group (Fig. 1B). SBP did not differ among groups (Fig. 1C). RVSP, the Fulton index (weight ratio of RV and (LV+ septum)), RV weight ratio (RV/BW), medial wall thickness, and the muscularization ratio of the periphery PA were significantly higher in all PH model groups than in controls (CH, MCT, MCT/CH group versus control group: p<0.05). Furthermore, these parameters were significantly higher in the combination MCT/CH group than in either the CH (p<0.05) or MCT (p<0.05) groups (Figs. 1D-H). After longer periods of exposure, rats in the MCT/CH group exhibited further increases in RVSP and in the Fulton index (RVSP: from 70 ± 10.8 mm Hg at 4 weeks to 75 ± 2.1 mm Hg at 5 weeks, and Fulton index: from 53.5 ± 7.5% at 4 weeks to 60.6 ± 6.5% at 5 weeks). These data suggested that the combination of MCT and CH methods (MCT/CH) increased severity of PH when compared to any single method (CH or MCT) in rats after 3 weeks.

**Fig 2:**
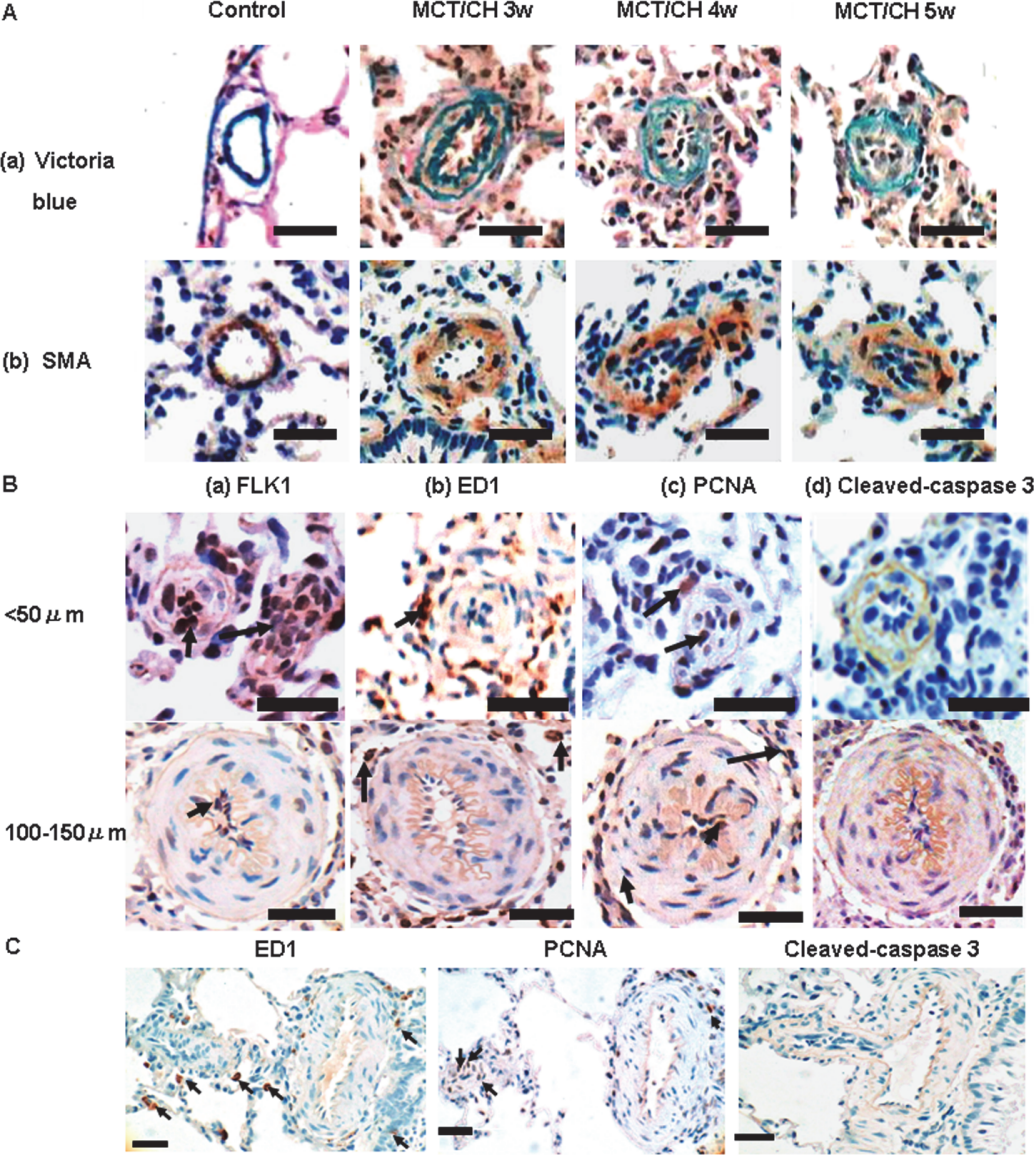
Morphological and immunohistochemical analysis of pulmonary arteries (PAs) in severe PH rats. MCT/CH resulted in vascular occlusive neointimal lesions. (A) Victoria blue staining and α-smooth muscle actin (SMA) immunohistochemical staining were used to delineate the elastic membrane and media of PAs in control and MCT/CH rats at 3 weeks (MCT/CH 3w), 4 weeks (MCT/CH 4w), and 5 weeks (MCT/CH 5w). (B) Immunohistochemical staining for (a) fetal liver kinase 1 (FLK1), (b) ED1, (c) proliferating cell nuclear antigen (PCNA) and (d) cleaved caspase-3 in lung tissue sections (arrows) from rats with severe PH (sections from control rats are not shown). Scale bar, 50 μm. (C) Occlusive neointimal lesions occurred distal to the branch points of small muscularized PAs, and showed positive ED1 and PCNA staining. Scale bar, 50 μm.

**Pulmonary artery (PA) morphology of MCT/CH rats indicated severe PH.** Lung tissue sections of MCT/CH rats were stained with Victoria blue (Fig. 2A-a) to visualize external and internal elastic membranes. They were immunohistochemically stained with SMA (Fig. 2A-b) to visualize the smooth muscle cells of the PA. In addition to increased medial wall thickness and muscularization ratio of small PAs, neointimal formation was also detected, and MCT/CH rats developed severe occlusions of the PA lumen at 4 weeks and 5 weeks.

Most neointimal lesions were located in the peripheral portions of the lung, with a PA diameter of approximately 50μm. To investigate the characteristics of these lesions, we used immunohistochemical staining to examine several markers. We found that fetal liver kinase 1 (FLk1)-positive endothelial cells occluded the vascular lumen (Fig. 2B-a), ED1-positive inflammatory cells were localized to the adventitia (Fig. 2B-b), PCNA-positive proliferating cells were distributed within the lumen and wall of the PA (Fig. 2B-c), while cleaved caspase-3-positive apoptotic cells were rarely detected (Fig. 2B-d).

Although similar marker expression patterns were observed in PAs with diameters ranging from 100 to 150μm, vascular remodeling was primarily localized to the medial wall, and less severe luminal occlusions were detected. However, occlusive neointimal lesions still occurred distal to the branch points of small muscularized PAs, and they were positive for ED1 and PCNA (Fig. 2C).

**Fig 3:**
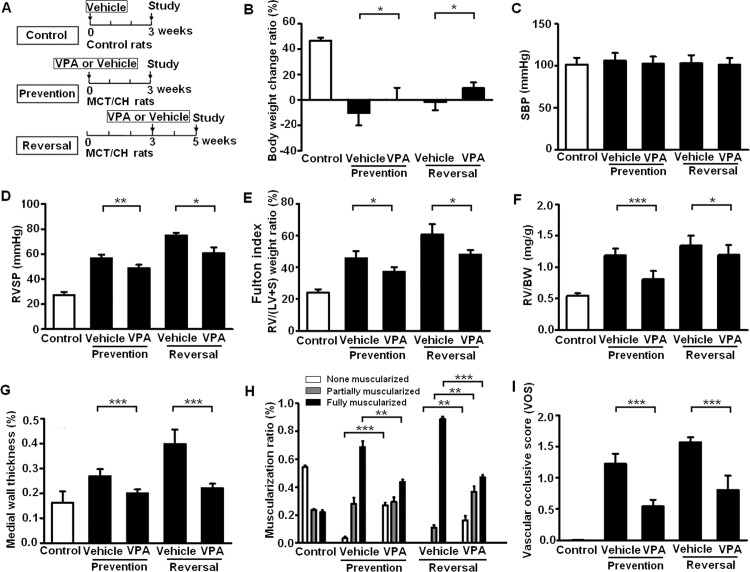
Therapeutic effects of valproic acid (VPA) on severe PH. (A) Schematic of therapeutic protocols used in the control, prevention, and reversal studies. The comparison of (B) body weight change ratio, (C) systemic blood pressure (SBP), (D) right ventricular systolic pressure (RVSP), (E) Fulton index, (F) the ratio of right ventricular weight to body weight (RV/BW), (G) medial wall thickness, (H) muscularization ratio and (I) the vascular occlusion score (VOS) of small PAs between the Vehicle-treated group and the VPA-treated group (n = 6 per group). * p < 0.05; ** p < 0.01; *** p < 0.001.

**Therapeutic effects of VPA on rats with MCT/CH-induced PH.** In the prevention study, MCT/CH rats were gavaged daily with VPA (300 mg/kg) or the Vehicle for 3 weeks (Fig. 3A). Compared with the Vehicle-treated group, the VPA-treated group had a significantly higher average body weight (Fig. 3B). No significant difference in SBP was observed (Fig. 3C), however, VPA treatment resulted in a significant improvement in RVSP (Fig. 3D), Fulton index (Fig. 3E), RV/BW (Fig. 3F), medial wall thickness (Fig. 3G), muscularization ratio (Fig. 3H) and the vascular occlusion score (VOS) (Fig. 3I) in peripheral PAs.

In the reversal study (Fig. 3A), MCT/CH rats received VPA (300 mg/kg) or the Vehicle via daily gavage from 3 to 5 weeks. Compared to the Vehicle-treated group, VPA treatment significantly increased body weight (Fig. 3B) and did not influence SBP (Fig. 3C). Similar to the prevention study, there was significant improvement in RVSP (Fig. 3D), Fulton index (Fig. 3E), RV/BW (Fig. 3F), medial wall thickness (Fig. 3G), muscularization ratio (Fig. 3H) and VOS (Fig. 3I) in peripheral PAs.

**Fig 4:**
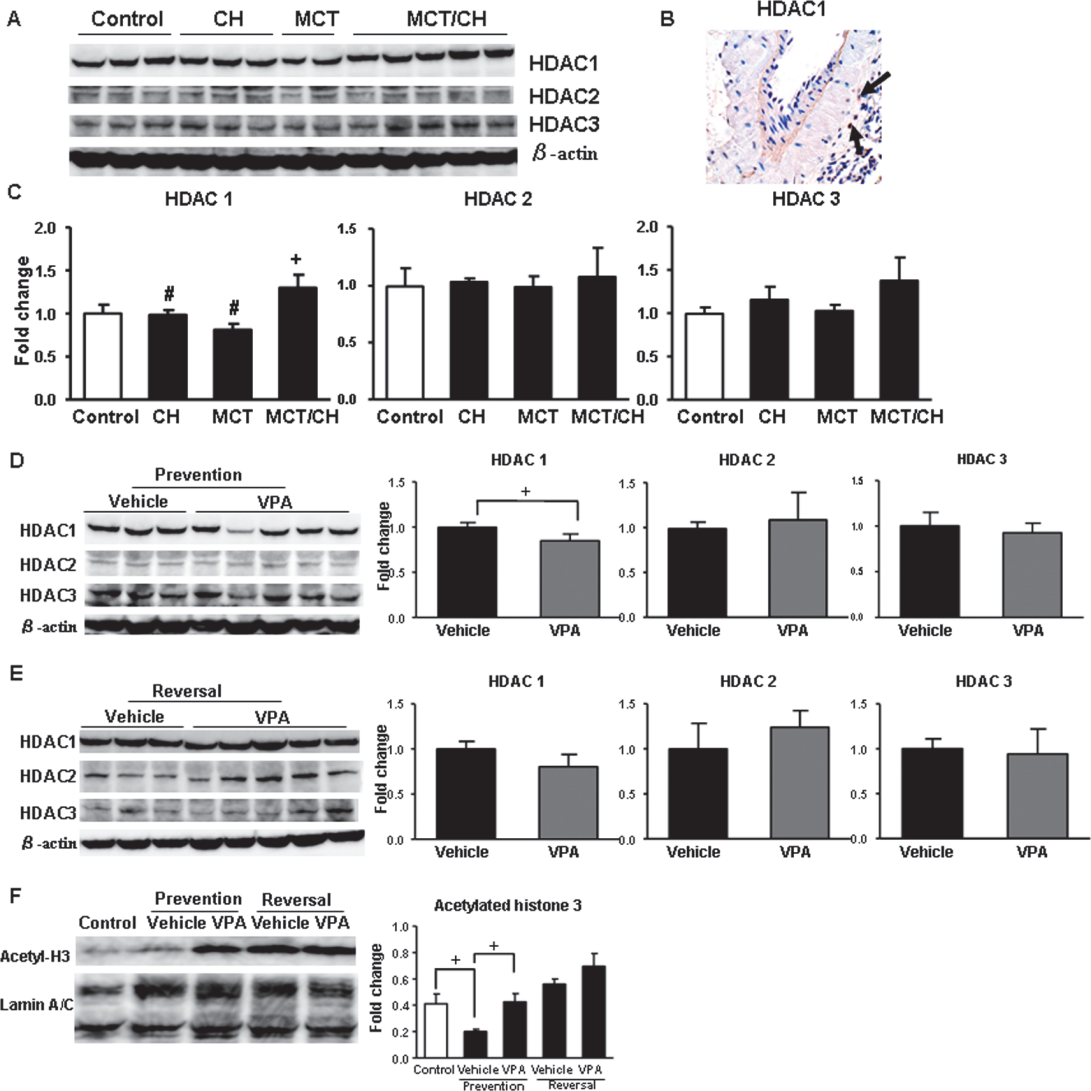
Histone deacetylase (HDAC) activity inhibition by VPA. (A) HDAC1, HDAC2, and HDAC3 expression levels in PH models were determined by western blot analysis. (B) Representative immunohistochemical staining of HDAC1 in MCT/CH rats. (C) Quantification of HDAC1, HDAC2 and HDAC3 expression in different PH model groups. Comparisons of HDAC1, HDAC2 and HDAC3 expression (D) in the prevention study and (E) the reversal study. (F) Acetylated-histone 3 expression in nuclear protein extracts. All western blots were quantified with a lumino-analyzer, and expression is shown as fold increases normalized to the expression of β-actin or lamin A/C. + p < 0.05 vs. control; # p < 0.05 vs. MCT/CH.

**Inhibition effects of VPA on HDAC activity in MCT/CH-induced severe PH.** The protein expression levels of HDAC1, HDAC2, and HDAC3 in lung tissues evaluated by western blotting in each PH model (Fig. 4A). HDAC1-positive cells were also observed in tissues of MCT/CH rats (Fig. 4B). Compared with either single method (CH or MCT), the combination method (MCT/CH) elevated HDAC1 expression significantly (CH vs. MCT/CH: P<0.05; MCT vs. MCT/CH: P<0.05), while HDAC2 and HDAC3 expression levels remained stable (Fig. 4C).

Compared with Vehicle-treated group, VPA treatment in the prevention study blocked the high expression of HDAC1 and did not significantly change the expression of HDAC2 and HDAC3 in MCT/CH rats (Fig. 4D). Increased expression of acetylated histone 3 has been reported to validate the inhibition of HDAC activity [5–8]. Nuclear proteins were extracted from lung tissues for western blotting analysis. VPA therapy significantly increased the expression of acetylated histone 3 in the prevention study (Fig. 4F). However, VPA treatment did not show significant regulation of HDAC (Fig. 4E) or acetylated histone 3 (Fig. 4F) expression in the reversal study.

**Fig 5:**
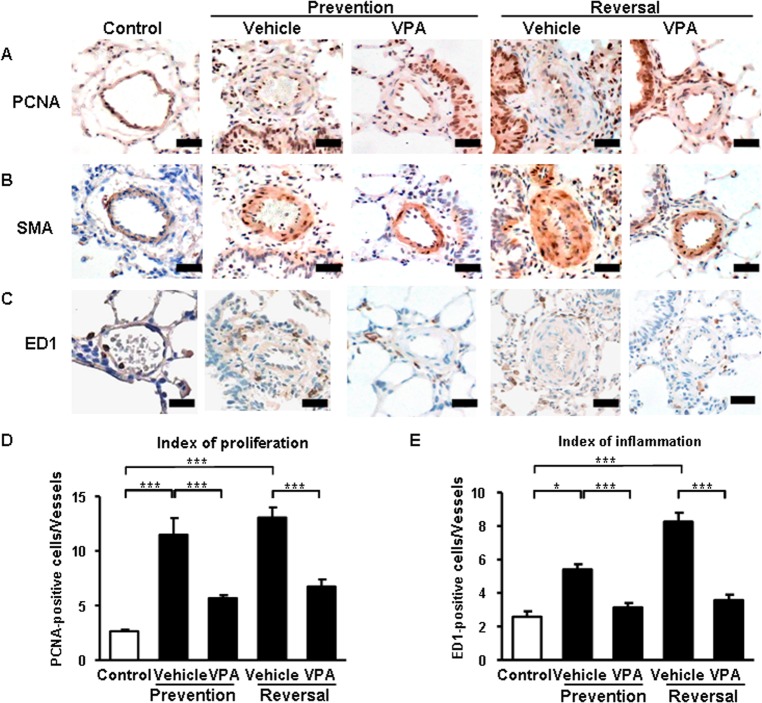
Effects of VPA on proliferation and inflammation. (A) PCNA, (B) SMA, and (C) ED1 immunohistochemical staining quantitative analysis of (D) the index of proliferation (the number of PCNA-positive cells per pulmonary vessel) and (E) the index of inflammation (the number of ED1-positive cells per pulmonary vessel) were used to show the regulation of proliferation and inflammation by VPA in the peripheral pulmonary vessels. Scale bar, 50 μm. * p < 0.05; *** p < 0.001.

**Effects of VPA on cell proliferation and inflammation.** The therapeutic effects of VPA on severe PH included reduced cell proliferation and inflammation in PA wall, as assessed by immunohistochemical staining. Compared to the control group, MCT/CH rats showed an increase in PCNA-positive proliferating cells (Figs. 5A-B and 5D) and ED1-positive inflammatory cells per PA (Figs. 5C and 5E) at both 3 weeks and 5 weeks. VPA treatment significantly reduced the indices of proliferation and inflammation in the PA not only in the prevention study, but also in the reversal study.

**Fig 6:**
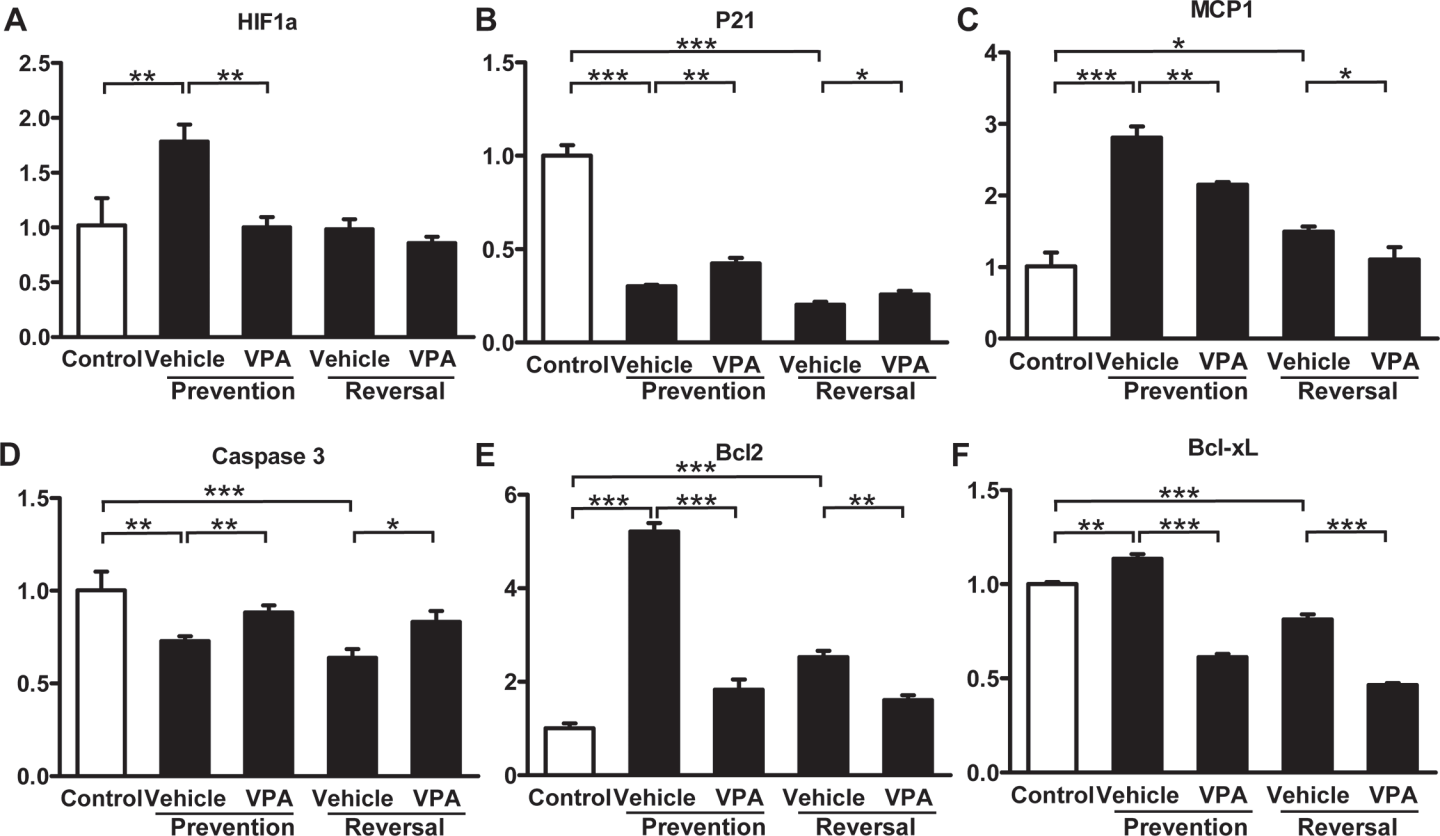
Effects of VPA on gene transcription. (A) *HIF1a*, (B) *P21*, (C) *MCP1*, (D) *Casp3*, (E) *Bcl2*, (F) *Bcl-xl* mRNA levels were assessed using real-time reverse transcription-polymerase chain reaction (RT-PCR) and are shown as fold change relative to the expression level of the control group. * p < 0.05; ** p < 0.01; *** p < 0.001. *Bcl2*, B-cell lymphoma 2; *Bcl-xl*, B-cell lymphoma-extra large; *MCP1*, monocyte chemoattractant protein 1; *HIF1a*, hypoxia induced factor 1a.

**Effects of VPA on gene transcription.** To determine the underlying molecular mechanisms modulating the therapeutic effects of VPA on rats with MCT/CH-induced severe PH, the PA was excised from the lower right lobe of the lung in each rat for total RNA preparation and quantitative PCR analysis. Rats with MCT/CH-induced severe PH expressed significantly higher levels of *HIF1a* (Fig. 6A), *MCP1* (Fig. 6C), *Bcl2* (Fig. 6E), and *Bcl-xl* (Fig. 6F), and they expressed lower levels of *p21* (Fig. 6B) and *Casp3* (Fig. 6D) at 3 weeks when compared to the control group. In the prevention study, VPA treatment led to a significant reduction in *HIF1a* (Fig. 6A), *MCP1* (Fig. 6B), *Bcl2* (Fig. 6E), and *Bcl-xl* (Fig. 6F) and elevation in *p21* (Fig. 6B) and *Casp3* (Fig. 6D) mRNA expression.

At 5 weeks, MCT/CH induced a significant increase in *MCP1* (Fig. 6C), *Bcl2* (Fig. 6E), and *Bcl-xl* (Fig. 6F) expression, and decreased *p21* (Fig. 6B) and *Casp3* (Fig. 6D) expression compared to the control group. In the reversal study, treatment with VPA decreased expression of *MCP1* (Fig. 6B), *Bcl2* (Fig. 6E), and *Bcl-xl* (Fig. 6F), while increasing the expression of *p21* (Fig. 6B) and *Casp3* (Fig. 6D). The expression of *HIF1a* also was decreased at 5 weeks when compared to 3 weeks in MCT/CH rats, however, VPA treatment did not show further attenuation of *HIF1a* expression at 5 weeks (Fig. 6A).

## Discussion

The major finding in the present study is that VPA, an inhibitor of class I HDACs, prevented and partially reversed the development of severe PH, induced by the combination of MCT and CH methods. Treatment with VPA reduced medial wall thickness, as well as the muscularization ratio and vascular occlusion score of small PAs, and it downregulated cell proliferation and inflammation within the vessel wall. The therapeutic effects of VPA in pulmonary vascular remodeling were also accompanied by a significant reduction in anti-apoptotic (Bcl2, Bcl-xl), inflammatory (MCP1) and hypoxia-stimulated (HIF1a) gene expression with a concomitant increase in apoptotic (Caspase3) and cell cycle-related (P21) gene expression.

Pathological features of pulmonary arterial hypertension (PAH) include medial hypertrophy, muscularization of nonmuscularized PA, and infiltration of inflammatory cells. In the advanced-stage of PAH, neointimal and complex lesions, such as the plexiform lesions, can form. Animal PH models recapitulating more features of human PAH are increasingly needed to investigate more effective pharmacological approaches. The CH and MCT models are two commonly used, classical PH models. However, single method is insufficient to induce progressive pulmonary vascular remodeling. As a result, CH model and MCT model are regarded as models for the less severe condition PH, and not PAH [11]. Since single method results in mild PH, researchers combined each method with other approaches to induce severe PH, such as the Sugen + hypoxia model [12] and MCT + pneumonectomy model [13]. Both hypoxia and MCT are essential factors involved in these severe PH models, and it was thought that the combining of MCT with CH could also induce severe vascular remodeling, including neointimal and plexiform lesions [6]. In the present study, this combination method (MCT/CH) resulted in enhanced RVSP, RV hypertrophy (Fulton index and RV weight ratio), and pulmonary vascular remodeling compared to that induced by any single method. Occlusive neointimal lesions were not detected in MCT or CH rats but were observed in MCT/CH rats (Fig. 2). These results indicated that MCT/CH-induced PH was more severe than CH or MCT single methods, and may be more similar to human PAH, although the classical phenotype of plexiform lesions was not apparent in the remodeled pulmonary vessels as reported [6].

HDACs decrease histone acetylation and play important roles in cell proliferation, inflammation in the cardiorenal axis [14]. Studies have also reported increased expression and activity of HDACs in certain cancers, and HDAC inhibitors show potential in suppressing cancer progression [15–17]. HDAC1 is a widely investigated member of the class I HDACs family, and it is implicated in the development of gastric, pancreatic, and hepatocellular cancers. Increased expression of HDAC1 has been observed in the lung tissues of PAH patients and in experimental rats with mild PH [5]. Our study demonstrated, for the first time, that rats with MCT/CH-induced severe PH exhibit elevated expression of HDAC1 but not HDAC2 or HDAC3 proteins. The CH method alone induced only a mild increase in HDAC1 expression, and MCT-induced PH resulted in a mild decrease, whereas the combination (MCT/CH) method showed significant elevation in HDAC1 protein levels (Figs. 4A-E). These results strongly suggest that HDAC1 has a potential role in the development of severe PH.

VPA is a mood stabilizer and anticonvulsant drug belonging to the short-chain fatty acid class of HDAC inhibitors with class I HDAC pharmacologic selectivity [18]. VPA inhibits HDAC activity and increases histone hyperacetylation in vivo and vitro, and it can induce differentiation of tumor cells [19]. VPA therapy induced beneficial effects in some experimental PH models [5,20]. In the present study, we showed that VPA therapy can prevent and partially reverse the development of severe PH in rats, as well as improve severe pulmonary vascular remodeling, including the reduction of occlusive neointimal lesions. Increased expression of HDAC1 in MCT/CH rats was inhibited by 15% in the prevention study and by 19% in the reversal study with VPA therapy. Furthermore, in the prevention study, the expression of acetylated histone 3 was upregulated by VPA at 3 weeks. Since the expression of acetylated histone 3 was already increased at 5 weeks, VPA therapy did not cause further elevation. These data suggested that VPA was effective against MCT/CH-induced severe PH, and its main mechanisms of action are HDAC1 inhibition and histone 3 acetylation.

Potential benefits of HDAC inhibition in cardiorenal diseases and cancer have been reported, and they may help explain the efficacy of VPA demonstrated in our study [21–22]. (1) HDAC inhibitors may reduce proinflammatory cytokine release and target mitogen-activated protein kinase phosphatase-1 [23], signal transducer and activator of transcription 1 [24], and nuclear factor κB [25] to modulate gene expression involved in inflammation. (2) HDAC inhibitors could increase bone morphogenetic protein (BMP) 7 expression, and suppress extracellular matrix production, cardiac fibroblasts activation and epithelial/endothelial-to-mesenchymal transition involved in anti-fibrotic activity [26]. (3) HDAC inhibitors have cardio-protective capability through suppression of B-type natriuretic peptide expression [27] and could modulate cell division involving p21 and FOXO3 genes [5] in experimental PH models. In the present study, VPA reduced the index of proliferation and expression of *HIF1a* (plays a role in hypoxia-induced cell proliferation), and upregulated CDK inhibitor *p21* expression. VPA reduced the index of inflammation and *MCP1* expression, and it promoted apoptosis, through increased expression of *Casp3* and decreased expression of *Bcl2* and *Bcl-xl*. These data revealed that beneficial effects of VPA in severe PH are due to its regulation of cell proliferation, inflammation, and apoptosis.

Taken together, these results confirmed the establishment of a severe PH model in rats using MCT/CH combination method and the therapeutic effects of VPA to prevent and partially reverse the development of severe PH. Through HDAC1 inhibition and histone 3 hyperacetylation, VPA regulates proliferation, inflammation, and apoptosis in remodeled pulmonary vessels. However, several limitations to this study should be acknowledged. First, 60mg/kg MCT is relatively high dose, exhibiting high mortality within 6 weeks. Further studies in rats may examine the effects of a reduced MCT dose combined with CH over longer observation periods. Second, VPA is not a specific inhibitor of HDAC1 and the effects of VPA on signaling pathways may also involve non-HDAC targets. Further studies using specific inhibitors of HDAC isoforms would be informative in this model of severe PH.

## Supporting Information

S1 ARRIVE Checklist The ARRIVE guidelines checklist. Following the ARRIVE (Animal Research: Reporting of In Vivo Experiments) guidelines, we designed and performed all our animal experiments, and prepared the manuscript.
(PDF)
